# Comment on: “Gene–Diet Interactions in Type 2 Diabetes: The Chicken and Egg Debate”

**DOI:** 10.3390/ijms20194878

**Published:** 2019-10-01

**Authors:** Duygu Onur Cura

**Affiliations:** Department of Molecular Medicine, Institute of Health Sciences, Dokuz Eylul University, 35330 Izmir, Turkey; duyguonur_05@hotmail.com; Tel.: +90-232-412-26-20

Ortega et al. published a comprehensive review which examines the relationship between nutritional genomics and Type 2 diabetes mellitus (T2DM) from a wide perspective [1]. The authors presented in detail the effect of nutrients on gene expression at the level of essential food components in the pathogenesis of T2DM as well as described the role of gene–diet interactions and epigenetic effects in T2DM.

Peroxisome proliferator activated receptors (PPARs) are a group of nuclear receptor subfamily which modulate energy metabolism and act as ligand activated transcription factors by small lipophilic molecules to regulate gene expression [2,3]. PPAR-γ (PPARG), a member of this family, has a key role in the glucose homeostasis and adipocyte differentiation. The *PPAR-γ* gene is localized in the chromosome 3p25.2 region. The most studied, prevalent and also benign Pro12Ala variant was identified in the PPARG2 isoform [4]. It is known that the T2DM risk is decreased in Ala allele carriers [5]. Ortega et al. emphasized that there was a correlation between high fat consumption and T2DM risk in individuals with ProPro genotype, but they did not mention fatty acid composition–gene interactions. A few saturated and unsaturated fatty acids are known to activate PPARs. PPAR-γ is known to exhibit a strong binding profile with polyunsaturated fatty acids (PUFAs) and a poor binding profile with monounsaturated fatty acids (MUFAs) [6]. In a study, it was shown that homeostatic model assessment for insulin resistance (HOMA-IR) values were higher in Ala carrier obese subjects who consumed low levels of MUFA [7]. It has been reported that there may be an increase in insulin resistance in individuals who consume a high saturated fatty acid (SFA) diet, even if they have the Ala allele [8]. In addition, the rs number given by Ortega et al. for this variant is incorrect. The rs number (rs180282) given for this variant both in Table 3 and in the text indicates an intronic variant of the guanine nucleotide-binding protein, gamma-transducing activity polypeptide 1 (*GNGT1*) gene localized in the region 7q21.3 [9]. The *PPAR-γ* gene is localized in the region 3p25.2 and the rs number of the Pro12Ala variant is rs1801282 [9].

Ortega et al. also mentioned gene–diet interactions related to variants of circadian related genes in T2DM. It is known that the circadian clock is involved in the control of metabolic functions and regulation of blood glucose concentration [10]. In an experimental study, it has been found that dysfunction of the circadian system in human pancreatic islet cells reduces insulin secretion [11]. The reference given for the *CLOCK* variant sampled in both text and Table 3 is incorrect. The authors cited a PREDIMED study by Corella et al. [12] investigating the relationship between the rs7903146 variant in the *TCF7L2* and obesity in T2DM. The correct reference seems to be another PREDIMED study performed by the same group [13]. The authors emphasized the interactions between dietary factors and *CLOCK* rs4580704, *CRY1* rs2287161 variants in T2DM, but other circadian related gene variants were not addressed. In a study investigating the gene–diet interactions of the *CLOCK* variants in metabolic syndrome patients, it was found that TT carriers of rs1801260 variant had higher insulin sensitivity, lower plasma insulin concentration and lower HOMA-IR after one year of low fat consumption [14]. Each 1% extra carbohydrate uptake has been shown to increase fasting glucose by 0.003 mmol/L in MTNR1B, rs1387153 variant T allele carriers [15]. 

Additionally, there is no information in the text about the PREDIMED study, which is associated with TCF7L2–diet interactions, given as reference incorrectly for the *CLOCK* variant. In this study, it was quoted that obesity status should be taken into consideration when classifying patients to obtain more accurate information about an interaction between diet and *TCF7L2* rs7903146 variant for T2DM risk [12].

This review successfully discussed the relationship between nutritional genomics and T2DM in light of current data in detail. Readers could obtain more accurate data with considering the aforementioned issues.
